# Changes in spirometry and pulmonary diffusing capacity in Mexican Hispanics approximately one year after having severe COVID-19: A dataset

**DOI:** 10.1016/j.dib.2024.110998

**Published:** 2024-10-10

**Authors:** Arturo Cortes-Telles, Gerald Stanley Zavorsky

**Affiliations:** a Respiratory Diseases Clinic, Regional High Specialty Hospital of the Yucatán Peninsula, IMSS-Wellness – Mérida, México; b Department of Physiology and Membrane Biology, Tupper Hall, University of California, Rm 4327 1275 Med Sciences Drive, Davis, CA 95616, United States

**Keywords:** COVID-19, SARS CoV-2, Spirometry, Lung diffusing capacity, Restriction, Airway obstruction

## Abstract

This observational longitudinal study was conducted at the Long-term follow-up COVID-19 Clinic in Mérida, Mexico, from March to August 2021. A total of 100 patients hospitalized for severe COVID-19 were enrolled. Inclusion criteria required participants to be adults over 18, recovering from severe COVID-19 as defined by the World Health Organization (oxygen saturation below 90 %, severe pneumonia, or signs of severe respiratory distress). Exclusion criteria included pneumonia from non-SARS-CoV-2 causes, mild or moderate COVID-19, or a single follow-up evaluation. Pulmonary function tests were conducted at approximately 100 and 400 days after diagnosis. The dataset includes 82 patients with baseline and follow-up spirometry, pulmonary diffusing capacity and alveolar volume. Morbidity history and fibrosis scores from high-resolution CT scans were also obtained. Finally, fitted z-scores for spirometry and pulmonary diffusing capacity were acquired from established reference equations. The freely accessible data (Version 4) is provided in both SPSS (.sav) and .csv format. at the Mendeley Data cloud-based repository and includes nominal data, ordinal data, and scalar data.

## Graphical abstract

**Figure:**
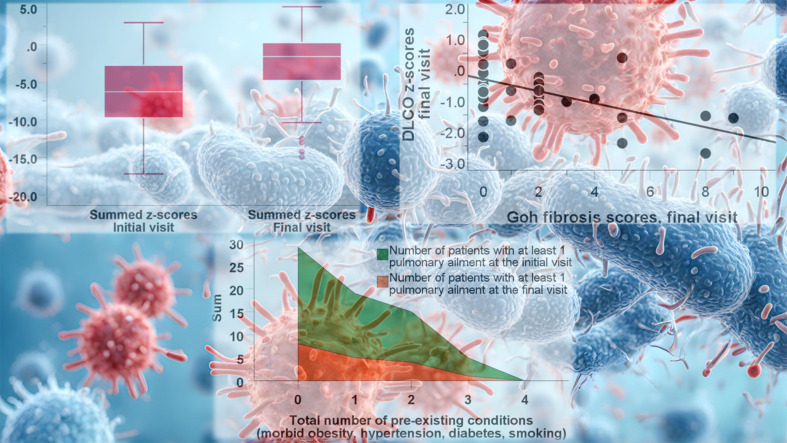

Specifications Table

**Table:** 

Subject	Health *and* Medical Sciences
Specific subject area	Pulmonary and Respiratory Medicine*.*
Type of data	Filtered data, 82 subjects, in SPSS (.sav) and .csv format
	
Data collection	Pulmonary function testing equipment was used to obtain spirometry and pulmonary diffusing capacity on two different occasions over an approximate one-year period. Lung function variables collected included spirometry measures, and pulmonary diffusing capacity, as well as pulmonary fibrosis scores from high resolution computed tomography (HRCT) scans. Z-scores were calculated for each patient based on normative data.
Data source location	Respiratory Diseases Clinic. Regional High Specialty Hospital of the Yucatán Peninsula, IMSS-Wellness – Mérida, México
Data accessibility	Repository name: Mendeley Data
	Data identification number: 10.17632/xmmyb9rgjj.4
	Direct URL to data: https://data.mendeley.com/datasets/xmmyb9rgjj/4
	This is the 4th version of the data. The clarity of the data is enhanced with more appropriate labelling.
	The data are in two formats:
	A) SPSS (.sav) format. Once the file is open you will find the labels for each parameter under the "VARIABLE VIEW" tab.
	B) .csv format.
Related research article	Cortes-Telles A., Solís-Díaz L.A., Mateos-Toledo H., Guenette J.A., Zavorsky G.S. (2024). Mexican Hispanics show significant improvement in lung function approximately one year after having severe COVID-19. Experimental Physiology doi:10.1113/EP091934.

## 1 Value of the Data

• It provides insights into long-term pulmonary outcomes in patients recovering from severe COVID-19, using z-scores and specific spirometric measures.
• It enables comparative studies on lung recovery trajectories across different populations, accounting for age, sex, comorbidities, and COVID-19 severity.
• It supports the development of predictive models for lung impairment, identifying high-risk patient profiles, and advancing personalized medicine in post-COVID-19 care.
• It serves as a benchmark for future research on long-term impacts of respiratory diseases beyond COVID-19, offering a baseline for longitudinal studies.
• It enhances meta-analyses by contributing detailed lung function and CT scan data, strengthening systematic reviews on post-COVID-19 outcomes.

## 2 Background

The motivation behind this dataset [1] was to address the gap in understanding the long-term pulmonary function outcomes in patients who have recovered from severe COVID-19. Given the novel nature of COVID-19 and its widespread impact, comprehensive data was needed to capture the recovery trajectory of lung function. This dataset [1] was generated using standardized spirometric and diffusing capacity measures expressed as z-scores to provide a detailed and quantitative assessment of lung recovery over time. The methodological framework involved longitudinal monitoring of patients, allowing for a robust analysis of pulmonary function changes post-recovery.

The theoretical background stems from the importance of understanding the potential long-term sequelae of COVID-19, particularly its effects on respiratory health. By providing data on lung function parameters and computed tomography scans, this dataset supports in-depth research into the recovery patterns and potential risks of persistent lung impairment.

This data article adds value by offering a comprehensive dataset that can be used for secondary analyses, comparative studies, and modeling efforts [1]. It enhances the primary findings of the original research [2] by providing the raw data needed to validate and expand upon those results, thereby supporting broader research initiatives in post-COVID-19 pulmonary health.

## 3 Data Description

The filtered data of 82 patients who had both initial and final pulmonary function tests are available in SPSS (.sav) format, which requires SPSS software to open. However, there is an identical data file in .csv format as well. Once the SPSS file is open, individuals can navigate to the “Variable View” tab. In this view, the “Label” column describes each parameter and its units (if applicable). For nominal variables, the “Values” column in the “Variable View” tab categorizes the variable. For example, “Sex” is a nominal variable; in the “Label” column, it is labelled as “Sex” (male or female). In the “Values” column, the coding is provided as 0 = female and 1 = male. The “Variable View” tab in the SPSS file offers a comprehensive explanation of each variable and its corresponding coding.

## 4 Materials and Methods

This dataset [1] includes all consecutive patients who were hospitalized and discharged due to severe COVID-19 between March 2021 and August 2021 and who attended their appointments to undergo a set of pulmonary function tests, including spirometry, lung diffusion, and a 6-min walk test, along with questionnaires aimed at assessing the persistence of symptoms. The first appointment was scheduled within 2 months of discharge, and subsequent evaluations were performed every 6 months. 100 patients were consecutively enrolled, but only 82 completed the pulmonary function tests at least twice, with a minimum of 1 year between the baseline and follow-up evaluations.

Pulmonary function tests were conducted following the standardized protocols outlined by the American Thoracic Society (ATS) and European Respiratory Society (ERS) [3] by utilizing z-scores to comprehensively assess the persistence and recovery of pulmonary abnormalities in the Mexican Hispanic population. Z-scores offer a more accurate interpretation of pulmonary function than the percent predicted values, as the lower limit of normal (LLN) for percent predicted varies with age [4,5]. The LLN for all lung function parameters was defined as a fitted z-score of –1.645 units (5th percentile). The upper limit of normal (ULN) was defined as a fitted z-score of +1.645 z-score units (95th percentile) – but the ULN was only pertinent for DLCO and KCO only because of the approach to its interpretation [3]. However, the dataset also displays the results when the LLN and ULN were defined as a z-score of –1.96 (2.5th percentile) and +1.96 (97.5th percentile) units, respectively [1].

Symptoms questionnaires were performed by the same evaluator, and all data was classified as positive/persistence =1 or absence=0. Symptoms included fatigue, shortness of breath on effort, cough, chest tightness, chest pain, sore throat, blocked and/or runny nose, loss of smell, loss of taste, diarrhea, abdominal pain, muscle or joint pain, headache, tachycardia, sore or red eyes, excessive sweating (including night sweats), hair loss, and weight loss.

A CT scan of the chest was performed within a median of 38 days of the final pulmonary function test. The CT images were evaluated by the same evaluator, who classified all abnormalities using the Goh score for fibrosis and inflammation [6].

Z-scores for forced expiratory volume in 1 s (FEV_1_), forced vital capacity (FVC), and FEV_1_/FVC ratio were calculated using Global Lung Function Initiative equations [7]. In contrast, pulmonary diffusing capacity (DLCO), alveolar volume (VA), and the carbon monoxide transfer coefficient (KCO) z-scores were calculated from Mexican-Hispanic reference equations [8]. Values below the LLN were considered abnormal. Paired *t*-tests and Wilcoxon signed-rank tests were used to compare pulmonary function changes between visits, depending on the distribution of z-scores. McNemar's test assessed changes in lung function proportions, with the Benjamini-Hochberg method applied to control for multiple comparisons [9].

The fitted z-scores for each of FEV_1_, FVC, FEV_1_/FVC ratio, DLCO, VA, and KCO were summed together to obtain one overall z-score value representing lung function. This overall summed z-score was obtained at the initial and the final visit. Linear regression was conducted to examine the relationship between changes in z-scores and time between tests. Analysis of covariance was used to compare improvements between sexes, adjusting for initial fitted z-scores.

Binary logistic regression assessed the association of variables like sex, age, BMI, pre-existing risk factors, and symptom changes with a meaningful improvement in z-scores. The criteria for determining a meaningful change in z-scores are detailed in Appendix A of the published article about this data [2].

Symptom counts were compared using Wilcoxon signed-rank tests, and Spearman's correlation assessed their association with z-score changes. The Goh fibrosis score was correlated with DLCO z-scores, with all imaging evaluated by the same radiologist for consistency.

IBM SPSS statistics (Version 29.0.1.0) and RStudio (Version 192 2024.04.2, build 764) was used for statistical analyses. A *p*-value of less than 0.05 was used for statistical significance.

## Limitations

The dataset – located in the online repository Mendeley Data [1] – has several limitations that should be acknowledged. First, only 44 out of the 82 patients underwent a CT scan during the final pulmonary function test (PFT). A key reason for the missing HRCT scans was that many patients had to return to work, making follow-up testing challenging. Second, there was a median gap of 38 days between the PFT and CT scan, as scheduling concurrent HRCT scans with PFTs proved difficult due to limited staffing and the availability of only one HRCT scanner. Third, there was variability in the timing of the two pulmonary evaluations, ranging from as few as 67 days to as many as 637 days between tests. Fourth, we were unable to consistently obtain hemoglobin measurements to adjust the DLCO results. However, since all patients resided at sea level and hemoglobin concentration generally does not significantly improve model fit in reference equations, this limitation is of minor concern. Lastly, the absence of pre-COVID-19 PFT results is a significant limitation, though using reference equations still allows for comparisons of pulmonary function against an unaffected cohort.

## Ethics Statement

This study was approved by the Ethics Committee of the Regional High Specialty Hospital of the Yucatán Peninsula, IMSS-Wellness – Mérida, México (Protocol number 2023–003). It was conducted in compliance with Clause 35 of the Helsinki Declaration. Informed consent for treatment and follow-up was obtained from all patients upon admission.

## CRediT Author Statement

G.S.Z = Conceptualization; Software; Writing of original draft; Formal analysis, Visualization A C-T. = Resources; Data Curation; Investigation, Methodology; Review and editing.

## Data Availability

Mendeley DataLong-term changes in spirometry and diffusing capacity in Mexican Hispanics with previous severe COVID-19 (Original data).
